# Supplementary material for: Genetic analysis of the septal peptidoglycan synthase FtsWI complex supports a conserved activation mechanism for SEDS-bPBP complexes
Source: PLoS Genet. 2021 Apr 15;17(4):e1009366. doi: 10.1371/journal.pgen.1009366 (PMC8078798; doi:10.1371/journal.pgen.1009366)
Supplement: S1 Text — (DOCX) [file pgen.1009366.s001.docx]

**Supplemental Information**

**Construction of strains**

LYA8

The strain LYA8 (W3110, *leu::Tn10 ftsI^K211I^*) was constructed by replacing the chromosomal *ftsI23* allele of PS413 (W3110, *leu::Tn10 ftsI*23) with the *ftsI^K211I^* allele by Lambda-Red recombineering as described by Datsenko KA, *et al.*(10). After electroporation of a DNA fragment containing the *ftsI^K211I^* coding sequence into PS413, recombinants were selected on LB plates with 1% NaCl at 42 °C. 10 recombinants were purified on the same selective medium. The presence of the mutation was verified by amplification and sequencing of the chromosomal ftsI DNA using primers 5-ftsI-seq and 3-ftsI-seq.

LYA9/pLY105

The strain LYA9/pLY105 (W3110, *leu::Tn10 ftsI^K211I^ ftsN::kan* / P_204_::*ftsI^K211I^*) was constructed by P1 transduction of the *ftsN::kan c*assette from CH34/pMG20 (TB28, *ftsN::kan*/P_BAD_:: *^ss^torA-bfp-ftsN^71-105^-le*) into strain LYA8. Transductants were selected on LB plates with 1% NaCl, 25μg/ml kanamycin, 1mM sodium citrate and 60 μM IPTG at 30, 37 and 42 °C. Transductants were only obtained at 42 °C and depended on the presence of IPTG for growth.

SD237

The strain SD237 was constructed by P1 transduction of the *ftsW::kan* cassette from EC912 [W3110, *ΔlacU169 gal-490 ftsW::kan*/pDSW406] into strain S3/pDSW406 (pBAD33, P_BAD_::*ftsW cat*). Transductants were selected on LB plates with 25 μg/ml kanamycin, 30 μg/ml chloramphenicol, 12.5 μg/ml tetracycline and sodium citrate at 30 °C and verified by arabinose-dependent growth and division.

SD285

The strain SD285 was constructed by P1 transduction of the *gfp-ftsI bla* cassette from EC436 [MC4100, Δ(*λattL-Lom*)::*bla lacI^q^* P_207_::gfp-ftsI)] into strain S3 (W3110, *leu::Tn10*). Transductants were selected on LB plates with 25μg/ml ampicillin and sodium citrate at 30 °C and verified by the localization of GFP-FtsI.

SD288

The strain SD288 was constructed by P1 transduction of the *ftsW::kan* cassette from EC912 [W3110, *ΔlacU169 gal-490 ftsW::kan*/pDSW406] into strain SD285/pSD257 (pSC101^ts^, *ftsW spc*). Transductants were selected on LB plates with 25μg/ml kanamycin, 25μg/ml spectinomycin, 25μg/ml ampicillin and sodium citrate at 30 °C and verified by temperature- sensitive growth and division.

SD292

The strain SD292 was constructed by P1 transduction of the *ftsW::kan* cassette from EC912 [W3110, *ΔlacU169 gal-490 ftsW::kan*/pDSW406] into strain W3110/pSD257 (pSC101^ts^, *ftsW spc*). Transductants were selected on LB plates with 25μg/ml kanamycin, 25μg/ml spectinomycin and sodium citrate at 30 °C and verified by temperature- sensitive growth and division.

SD295

The strain SD295 was constructed by P1 transduction of the *recA::Tn10* cassette from PB143 [PB103, *ftsZ^0^ recA::Tn10*/pCX41(pSC101^ts^, *ftsZ*)) into strain SD292. Transductants were selected on LB plates with 12.5μg/ml tetracycline, 25μg/ml kanamycin, 25μg/ml spectinomycin and sodium citrate at 30 °C and verified by sensitivity to UV radiation.

SD366

The strain SD366 was constructed by P1 transduction of the *ftsW::kan* cassette from EC912 [W3110, *ΔlacU169 gal-490 ftsW::kan*/pDSW406] into strain TB28/pSD257 (pSC101^ts^, *ftsW spc*). Transductants were selected on LB plates with 25μg/ml kanamycin, 25μg/ml spectinomycin and sodium citrate at 30 °C and verified by temperature- sensitive growth and division.

SD367

The strain SD367 was constructed by P1 transduction of the *ftsW::kan* cassette from EC912 [W3110, *ΔlacU169 gal-490 ftsW::kan*/pDSW406] into strain BL167/pSD257 [TB28, *ftsB^E56A^*/ pSD257 (pSC101^ts^, *ftsW spc*)]. Transductants were selected on LB plates with 25μg/ml kanamycin, 25μg/ml spectinomycin and sodium citrate at 30 °C and verified by temperature- sensitive growth and division.

SD387

The strain SD387 was constructed by P1 transduction of the *ftsW::kan* cassette from EC912 [W3110, *ΔlacU169 gal-490 ftsW::kan*/pDSW406] into strain SD221/pSD257 [W3110, *leu::Tn10 ftsA**/ pSD257 (pSC101^ts^, *ftsW spc*)]. Transductants were selected on LB + 0.2M sucrose plates with 25μg/ml kanamycin, 25μg/ml spectinomycin, 12.5μg/ml tetracycline and sodium citrate at 30 °C and verified by temperature- sensitive growth and presence of the ftsA* mutation by sequencing.

SD389

The strain SD389 was constructed by P1 transduction of the *ftsW::kan ftsA** cassette from SD387 into strain W3110/pSD257 (pSC101^ts^, *ftsW spc*)]. Transductants were selected on LB plates with 25μg/ml kanamycin, 25μg/ml spectinomycin and sodium citrate at 30 °C and verified by temperature- sensitive growth, sensitivity to tetracycline and the presence of the ftsA* mutation by sequencing.

SD390

The strain SD390 was constructed by P1 transduction of the *recA::Tn10* cassette from PB143 [PB103, *ftsZ^0^ recA::Tn10*/pCX41(pSC101^ts^, *ftsZ*)) into strain SD389. Transductants were selected on LB plates with 25μg/ml kanamycin, 25μg/ml spectinomycin 12.5μg/ml tetracycline and sodium citrate at 30 °C and verified by temperature- sensitive growth and sensitivity to UV radiation.

SD399

The strain SD399 was constructed by P1 transduction of the *ftsL::kan* cassette from BL156 (TB28, ftsL::kan) into strain W3110/pSD296 (pBAD33, P_BAD_::*ftsL* cat). Transductants were selected on LB plates with 30 μg/ml chloramphenicol, 25μg/ml kanamycin and sodium citrate at 30 °C and verified by arabinose-dependent growth and division.

SD488

The strain SD488 was constructed by replacing the chromosomal *ftsW^WT^* allele of S3 with the *ftsW^E289G^* allele from plasmid pSD257-E289G (pSC101^ts^, *ftsW^E289G^*) by the methods of Hamilton et al. The presence of the mutation was verified by amplification and sequencing of the chromosomal DNA using primers 5-ftsW-seq and 3-ftsW-seq.

SD530

The strain SD530 was constructed by P1 transduction of the *ftsN::kan c*assette from CH34/pMG20 (TB28, *ftsN::kan*/P_BAD_:: *^ss^torA-bfp-ftsN^71-105^-le*) into strain SD488. Transductants were selected on LB plates with 25μg/ml kanamycin and sodium citrate at 37 °C.

SD531

The strain SD531 was constructed by P1 transduction of the *ftsN::kan c*assette from CH34/pMG20 (TB28, *ftsN::kan*/P_BAD_:: *^ss^torA-bfp-ftsN^71-105^-le*) into strain W3110/pSEB417 (pDSW208, P_trc_::*ftsN bla*). Transductants were selected on LB plates with 25μg/ml kanamycin, 25μg/ml ampicillin and sodium citrate at 37 °C.

SD532

The strain SD532 was constructed by P1 transduction of the *ftsN::kan c*assette from CH34/pMG20 (TB28, *ftsN::kan*/P_BAD_:: *^ss^torA-bfp-ftsN^71-105^-le*) into strain W3110/pSEB429-M269I (pDSW208, P_trc_::*ftsW^M269I^ bla*). Transductants were selected on LB plates with 25μg/ml kanamycin, 25 μg/ml ampicillin, 60 μM IPTG and sodium citrate at 37 °C.

SD533

The strain SD533 was constructed by P1 transduction of the *ftsN::kan c*assette from CH34/pMG20 (TB28, *ftsN::kan*/P_BAD_:: *^ss^torA-bfp-ftsN^71-105^-le*) into strain SD247/pSEB417 [W3110, *leu::Tn10 ftsW^M269I^* /(pDSW208, P_trc_::*ftsN bla*). Transductants were selected on LB plates with 25μg/ml kanamycin, 25μg/ml ampicillin and sodium citrate at 37 °C.

SD534

The strain SD534 was constructed by P1 transduction of the *ftsN::kan c*assette from CH34/pMG20 (TB28, *ftsN::kan*/P_BAD_:: *^ss^torA-bfp-ftsN^71-105^-le*) into strain SD247/pSEB429-M269I [W3110, *leu::Tn10 ftsW^M269I^ /*(pDSW208, P_trc_::*ftsW^M269I^ bla*)]. Transductants were selected on LB plates with 25μg/ml kanamycin, 25 μg/ml ampicillin, 60 μM IPTG and sodium citrate at 37 °C.

**Construction of plasmids**

pLY91

The plasmid pLY91 (pDSW208, P*_204_*::*ftsI*) was constructed by ligation of an EcoRI/HindIII digested DNA fragment containing *ftsI* into pDSW208 digested with the same enzymes. The DNA fragment was amplified from W3110 chromosomal DNA using primers pLY91-I-F and pLY91-I-R.

PLY107

The plasmid pLY107 (pBAD33, P*_BAD_*::*ftsI*) was constructed by ligation of an XbaI/HindIII digested DNA fragment containing *ftsI* into pBAD33 digested with the same enzymes. The DNA fragment was amplified from W3110 chromosomal DNA using primers pLY107-I-F and pLY107-I-R.

pLY123

The plasmid pLY123 (pKT25, P*_lac_*::t25-*ftsL*) was constructed by ligation of an BamHI/EcoRI digested DNA fragment containing *ftsL* into pKT25 digested with the same enzymes. The DNA fragment was amplified from W3110 chromosomal DNA using primers pLY123-BamHI-F and pLY123-EcoRI-R.

pLY124

The plasmid pLY124 (pKT25, P*_lac_*::t25-*ftsB*) was constructed by ligation of an BamHI/EcoRI digested DNA fragment containing *ftsB* into pKT25 digested with the same enzymes. The DNA fragment was amplified from W3110 chromosomal DNA using primers pLY124-BamHI-F and pLY124-EcoRI-R.

pSD296

The plasmid PSD296 (pBAD33, P*_BAD_*::*ftsL*) was constructed by ligation of an XbaI/HindIII digested DNA fragment containing *ftsL* into pBAD33 digested with the same enzymes. The DNA fragment was amplified from plasmid pSD256 (pSC101ts, P_syn_::*ftsL spc*) using primers 5-XbaI-ftsL and 3-ftsL-HindIII.

pSD348

The plasmid pSD348 (pDSW210, P*_206_*::*l60*-*gfp bla*) was constructed by ligation of an EcoRI/HindIII digested DNA fragment containing *l60*-*gfp* from pSD286 (pDSW208, P*_204_*::*l60*-*gfp bla*) into pDSW210 digested with the same enzymes.

pSD349

The plasmid pSD348 (pDSW210, P*_206_*::ftsW-*l60*-*gfp bla*) was constructed by ligation of an EcoRI/XbaI digested DNA fragment containing *ftsW* into pSD348 digested with the same enzymes. The DNA fragment was amplified from plasmid pSEB429 (pDSW208, P*_204_*::*ftsW bla*) using primers 5-EcoRI-ftsW and 3-ftsW-XbaI.

pUT18C-ftsQ

The plasmid pUT18C-ftsQ (pUT18C, P*_lac_*::t18-*ftsQ*) was constructed by ligation of an XbaI/EcoRI digested DNA fragment containing *ftsQ* into pUT18C digested with the same enzymes. The DNA fragment was amplified from chromosomal DNA using primers 5-XbaI-ftsQ and 3-ftsQ-EcoRI.

pUT18C-ftsI

The plasmid pUT18C-ftsI (pUT18C, P*_lac_*::t18-*ftsI*) was constructed by ligation of a BamHI/EcoRI digested DNA fragment containing *ftsI* into pUT18C digested with the same enzymes. The DNA fragment was amplified from chromosomal DNA using primers 5-BamHI-ftsI and 3-ftsI-EcoRI.

Variants of plasmids carrying different *ftsW* or *ftsL* mutations were created by site-directed mutagenesis using indicated plasmids and primers.
