# Supplementary material for: Genetic analysis of the septal peptidoglycan synthase FtsWI complex supports a conserved activation mechanism for SEDS-bPBP complexes
Source: PLoS Genet. 2021 Apr 15;17(4):e1009366. doi: 10.1371/journal.pgen.1009366 (PMC8078798; doi:10.1371/journal.pgen.1009366)
Supplement: S1 Table — (DOCX) [file pgen.1009366.s002.docx]

**Supplemental Information**

**S1 Table. Bacterial strains used in this study**

| Strain | Genotype | Source /Reference |
| --- | --- | --- |
| BTH101 | *cya-99 araD139 galE15 galK16 rpsL1 hsdR2 mcrA1 mcrB1* | (1) |
| CH34/pMG20 | TB28, *ftsN::kan*/ (pSC101ts, ftsA) | (2) |
| JS238 | MC1061 malPp::lacIQ srlC::Tn10 *recA1* | (3) |
| LYA8 | W3110, *leu::Tn10 ftsI^K211I^* | This study |
| LYA9/pLY105 | W3110, *leu::Tn10 ftsI^K211I^ ftsN::kan /P_204_::ftsI^K211I^* | This study |
| MCI23ΔrecA | MC4100, *ftsI23, recA::spec* | (4) |
| S3 | W3110, *leu::Tn10* | (5) |
| SD237 | W3110, *ftsW::kan* / pDSW406 | This study |
| SD247 | S3, *ftsW^M269I^* | (6) |
| SD264 | S3, *ftsN::kan /* pBL154 | (6) |
| SD265 | S3, *ftsW^M269I^ ftsN::kan /pBL154* | (6) |
| SD285 | W3110, *leu::Tn10* P_trc_*::gfp-ftsI, bla* | This study |
| SD288 | W3110, *leu::Tn10 ftsW::kan* P_trc_*::gfp-ftsI, bla* / pSD257 | This study |
| SD292 | W3110, *ftsW::kan* / pSD257 | This study |
| SD295 | W3110, *ftsW::kan recA::Tn10* / pSD257 | This study |
| SD366 | TB28, *ftsW::kan* / pSD257 | This study |
| SD367 | TB28, *ftsW::kan ftsB^E56A^*/ pSD257 | This study |
| SD390 | W3110, *ftsA* ftsW::kan recA::Tn10* / pSD257 | This study |
| SD399 | W3110, *ftsL::kan /* pSD256 | This study |
| SD488 | S3, *ftsW^E289G^* | This study |
| SD530 | S3, *ftsW^E289G^ ftsN::kan* | This study |
| SD531 | W3110, *ftsN::kan /* pSEB417 | This study |
| SD532 | W3110, *ftsN::kan ftsW^M269I^/* pSEB429-M269I | This study |
| SD533 | W3110, *ftsN::kan ftsW^M269I^/* pSEB417 | This study |
| SD534 | W3110, *ftsN::kan ftsW^M269I^/* pSEB429-M269I | This study |
| W3110 | *F^-^ lambda^-^ IN (rrnD-rrnE)1 rph-1* | Lab collection |

**References**:

1. Karimova G, Dautin N, Ladant D. Interaction network among Escherichia coli membrane proteins involved in cell division as revealed by bacterial two-hybrid analysis. J Bacteriol. 2005;187(7):2233-43.

2. Gerding MA, Liu B, Bendezu FO, Hale CA, Bernhardt TG, de Boer PA. Self-enhanced accumulation of FtsN at Division Sites and Roles for Other Proteins with a SPOR domain (DamX, DedD, and RlpA) in Escherichia coli cell constriction. J Bacteriol. 2009;191(24):7383-401.

3. Pichoff S, Vollrath B, Touriol C, Bouche JP. Deletion analysis of gene minE which encodes the topological specificity factor of cell division in Escherichia coli. Mol Microbiol. 1995;18(2):321-9.

4. Park KT, Du S, Lutkenhaus J. Essential Role for FtsL in Activation of Septal Peptidoglycan Synthesis. mBio. 2020;11(6).

5. Shen B, Lutkenhaus J. The conserved C-terminal tail of FtsZ is required for the septal localization and division inhibitory activity of MinC(C)/MinD. Mol Microbiol. 2009;72(2):410-24.

6. Du S, Pichoff S, Lutkenhaus J. FtsEX acts on FtsA to regulate divisome assembly and activity. Proc Natl Acad Sci U S A. 2016;113(34):E5052-61.
