# Supplementary material for: Genetic analysis of the septal peptidoglycan synthase FtsWI complex supports a conserved activation mechanism for SEDS-bPBP complexes
Source: PLoS Genet. 2021 Apr 15;17(4):e1009366. doi: 10.1371/journal.pgen.1009366 (PMC8078798; doi:10.1371/journal.pgen.1009366)
Supplement: S2 Table — (DOCX) [file pgen.1009366.s003.docx]

**Supplemental Information**

**S2 Table. Plasmids used in this study**

| Plasmid | Genotype | Source /Reference |
| --- | --- | --- |
| pBL154 | pSC101^ts^*, aadA repA^ts^* P_syn135_::*ftsN* | (1) |
| pDSW406 | pBAD33, cat P_BAD_::*ftsW* | (2) |
| pDSW406-M269K | pBAD33, cat P_BAD_::*ftsW^M269K^* | This study |
| pDSW406-A270T | pBAD33, cat P_BAD_::*ftsW^A270T^* | This study |
| pKT25 | pACYC184, kan P_lac_::t25- | (3) |
| pKT25-ftsW | pACYC184, kan P_lac_::t25-ftsW | (3) |
| pLY81 | pDSW208, bla P_204_::ftsW^M269V^ | This study |
| pLY82 | pDSW208, bla P_204_::ftsW^M269F^ | This study |
| pLY83 | pDSW208, bla P_204_::ftsW^M269A^ | This study |
| pLY85 | pDSW208, bla P_204_::ftsW^M269E^ | This study |
| pLY87 | pDSW208, bla P_204_::ftsW^E289R^ | This study |
| pLY88 | pDSW208, bla P_204_::ftsW^E289L^ | This study |
| pLY89 | pDSW208, bla P_204_::ftsW^E289Q^ | This study |
| pLY91 | pDSW208, bla P_204_::ftsI | This study |
| pLY105 | pDSW208, bla P_204_::ftsI^K211I^ | This study |
| pLY107 | pBAD33, cat P_BAD_::ftsI | This study |
| pLY110 | pBAD33, cat P_BAD_::ftsI^L62P^ | This study |
| pLY111 | pBAD33, cat P_BAD_::ftsI^R210C^ | This study |
| pLY116 | pDSW208, bla P_204_::ftsI^K211A^ | This study |
| pLY117 | pDSW208, bla P_204_::ftsI^K211T^ | This study |
| pLY118 | pDSW208, bla P_204_::ftsI^K211F^ | This study |
| pLY119 | pDSW208, bla P_204_::ftsI^K211E^ | This study |
| pLY123 | pKT25, kan P_lac_::t25-ftsL | This study |
| pLY124 | pKT25, kan P_lac_::t25-ftsB | This study |
| pLY125 | pUC18C, bla P_lac_::t18-ftsI^K211I^ | This study |
| pLY129 | pDSW208, bla P_204_::ftsI^L62P,K211I^ | This study |
| pLY130 | pDSW208, bla P_204_::ftsI^R210C,K211I^ | This study |
| pLY133 | pDSW208, bla P_204_::ftsI^L62P^ | This study |
| pLY134 | pDSW208, bla P_204_::ftsI^R210C^ | This study |
| pLY135 | pBAD33, cat P_BAD_::ftsI^G57D^ | This study |
| pLY136 | pBAD33, cat P_BAD_::ftsI^S61F^ | This study |
| pLY137 | pDSW208, bla P_204_::ftsI^G57D^ | This study |
| pLY138 | pDSW208, bla P_204_::ftsI^S61F^ | This study |
| pLY139 | pDSW208, bla P_204_::ftsI^G57D,K211I^ | This study |
| pLY140 | pDSW208, bla P_204_::ftsI^S61F,K211I^ | This study |
| pMG20 | pBAD33, cat P_BAD_::^ss^torA-bfp-ftsN^71-105^-le | (4) |
| pSD221 | pEXT22, kan P_tac_::ftsEX | (5) |
| pSD221-D162N | pEXT22, kan P_tac_::ftsE^D162N^X | (5) |
| pSD256 | pSC101^ts^*, aadA repA^ts^* P_ftsL_::ftsL | (5) |
| pSD257 | pSC101^ts^*, aadA repA^ts^* P_ftsW_::ftsW | (5) |
| pSD257-E289G | pSC101^ts^*, aadA repA^ts^* P_ftsW_::ftsW^E289G^ | This study |
| pSD296 | pBAD33, cat P_BAD_::ftsL | (6) |
| pSD348 | pDSW210, bla P_206_::CcftsZlinker60-gfp | This study |
| pSD349 | pDSW210, bla P_206_::ftsW-l60-gfp | This study |
| pSD349-M269K | pDSW210, bla P_206_::ftsW^M269K^-l60-gfp | This study |
| pSD349-A270T | pDSW210, bla P_206_::ftsW^A270T^-l60-gfp | This study |
| pSEB417 | pDSW208, bla P_204_::ftsN | (7) |
| pSEB429 | pDSW208, bla P_204_::ftsW | (7) |
| pSEB429-M269I | pDSW208, bla P_204_::ftsW^M269I^ | This study |
| pSEB429-M269K | pDSW208, bla P_204_::ftsW^M269K^ | This study |
| pSEB429-A270T | pDSW208, bla P_204_::ftsW^A270T^ | This study |
| pSEB429-E289G | pDSW208, bla P_204_::ftsW^E289G^ | This study |
| pSEB429-ME/KG | pDSW208, bla P_204_::ftsW^M269K,E289G^ | This study |
| pSEB429-AE/KG | pDSW208, bla P_204_::ftsW^A270T,E289G^ | This study |
| pUT18C | pMB1, bla P_lac_::t18- | (3) |
| pUT18C-ftsB | pMB1, bla P_lac_::t18-ftsB | (3) |
| pUT18C-ftsL | pMB1, bla P_lac_::t18-ftsL | (3) |
| pUT18C-ftsQ | pMB1, bla P_lac_::t18-ftsQ | This study |
| pUT18C-ftsI | pMB1, bla P_lac_::t18-ftsI | This study |

**References**:

1. Liu B, Persons L, Lee L, de Boer PA. Roles for both FtsA and the FtsBLQ subcomplex in FtsN-stimulated cell constriction in Escherichia coli. Mol Microbiol. 2015;95(6):945-70.

2. Mercer KL, Weiss DS. The Escherichia coli cell division protein FtsW is required to recruit its cognate transpeptidase, FtsI (PBP3), to the division site. J Bacteriol. 2002;184(4):904-12.

3. Karimova G, Dautin N, Ladant D. Interaction network among Escherichia coli membrane proteins involved in cell division as revealed by bacterial two-hybrid analysis. J Bacteriol. 2005;187(7):2233-43.

4. Gerding MA, Liu B, Bendezu FO, Hale CA, Bernhardt TG, de Boer PA. Self-enhanced accumulation of FtsN at Division Sites and Roles for Other Proteins with a SPOR domain (DamX, DedD, and RlpA) in Escherichia coli cell constriction. J Bacteriol. 2009;191(24):7383-401.

5. Du S, Pichoff S, Lutkenhaus J. FtsEX acts on FtsA to regulate divisome assembly and activity. Proc Natl Acad Sci U S A. 2016;113(34):E5052-61.

6. Park KT, Du S, Lutkenhaus J. Essential Role for FtsL in Activation of Septal Peptidoglycan Synthesis. mBio. 2020;11(6).

7. Pichoff S, Du S, Lutkenhaus J. The bypass of ZipA by overexpression of FtsN requires a previously unknown conserved FtsN motif essential for FtsA-FtsN interaction supporting a model in which FtsA monomers recruit late cell division proteins to the Z ring. Mol Microbiol. 2015;95(6):971-87.
