# Supplementary material for: Genetic analysis of the septal peptidoglycan synthase FtsWI complex supports a conserved activation mechanism for SEDS-bPBP complexes
Source: PLoS Genet. 2021 Apr 15;17(4):e1009366. doi: 10.1371/journal.pgen.1009366 (PMC8078798; doi:10.1371/journal.pgen.1009366)
Supplement: S3 Table — (DOCX) [file pgen.1009366.s004.docx]

**Supplemental Information**

**S3 Table. Primers used in this study**

| Primer name | Sequence |
| --- | --- |
| ftsI-G57D-F | GATATGCTGGTGAAAGAG**GAC**GACATGCGTTCTCTTC |
| ftsI-G57D-R | GAAGAGAACGCATGTC**GTC**CTCTTTCACCAGCATATC |
| ftsI-S61F-F | GAGGGCGACATGCGT**TTT**CTTCGCGTTCAGCAAG |
| ftsI-S61F-R | CTTGCTGAACGCGAAG**AAA**ACGCATGTCGCCCTC |
| ftsI-L62P-F | GGCGACATGCGTTCT**CCT**CGCGTTCAGCAAGTTTC |
| ftsI-L62P-R | GAAACTTGCTGAACGCG**AGG**AGAACGCATGTCGCC |
| ftsI-R210C-F | GGTGAGCGCATTGTG**TGT**AAAGACCGCTATGGTC |
| ftsI-R210C-R | GACCATTAGCGGTCTTT**ACA**CACAATGCGCTCACC |
| ftsI-K211I-F | GAGCGCATTGTGCGT**ATA**GACCGCTATGGTCGC |
| ftsI-K211I-R | GCGACCATAGCGGTC**TAT**ACGCACAATGCGCTC |
| ftsI-K211A-F | GAGCGCATTGTGCGT**GCA**GACCGCTATGGTCGC |
| ftsI-K211A-R | GCGACCATAGCGGTC**TGC**ACGCACAATGCGCTC |
| ftsI-K211T-F | GAGCGCATTGTGCGT**ACA**GACCGCTATGGTCGC |
| ftsI-K211T-R | GCGACCATAGCGGTC**TGT**ACGCACAATGCGCTC |
| ftsI-K211F-F | GAGCGCATTGTGCGT**TTC**GACCGCTATGGTCGC |
| ftsI-K211F-R | GCGACCATAGCGGTC**GAA**ACGCACAATGCGCTC |
| ftsI-K211E-F | GAGCGCATTGTGCGT**GAA**GACCGCTATGGTCGC |
| ftsI-K211E-R | GCGACCATAGCGGTC**TTC**ACGCACAATGCGCTC |
| ftsI-R210C,K211I-F | GGTGAGCGCATTGTG**TGTATA**GACCGCTATGGTCGC |
| ftsI-R210C,K211I-R | GCGACCATAGCGGTC**TATACA**CACAATGCGCTCACC |
| ftsW-M269I-F | GTTAACGCAATCGCTG**ATA**GCGTTTGGTCGCGGCG |
| ftsW-M269I-R | CGCCGCGACCAAACGC**TAT**CAGCGATTGCGTTAAC |
| ftsW-M269V-F | GTTAACGCAATCGCTG**GTG**GCGTTTGGTCGCGGCG |
| ftsW-M269V-R | CGCCGCGACCAAACGC**CAC**CAGCGATTGCGTTAAC |
| ftsW-M269F-F | GTTAACGCAATCGCTG**TTC**GCGTTTGGTCGCGGCG |
| ftsW-M269F-R | CGCCGCGACCAAACGC**GAA**CAGCGATTGCGTTAAC |
| ftsW-M269A-F | GTTAACGCAATCGCTG**GCG**GCGTTTGGTCGCGGCG |
| ftsW-M269A-R | CGCCGCGACCAAACGC**CGC**CAGCGATTGCGTTAAC |
| ftsW-M269K-F | GTTAACGCAATCGCTG**AAG**GCGTTTGGTCGCGGCG |
| ftsW-M269K-R | CGCCGCGACCAAACGC**CTT**CAGCGATTGCGTTAAC |
| ftsW-M269E-F | GTTAACGCAATCGCTG**GAG**GCGTTTGGTCGCGGCG |
| ftsW-M269E-R | CGCCGCGACCAAACGC**CTC**CAGCGATTGCGTTAAC |
| ftsW-A270T-F | GTTAACGCAATCGCTGATG**ACG**TTTGGTCGCGGCGAAC |
| ftsW-A270T-R | GTTCGCCGCGACCAAA**CGT**CATCAGCGATTGCGTTAAC |
| ftsW-E289G-F | CTCGGTACAAAAACTG**GGG**TATCTGCCGGAAGCG |
| ftsW-E289G-R | CGCTTCCGGCAGATA**CCC**CAGTTTTTGTACCGAG |
| ftsW-E289R-F | CTCGGTACAAAAACTG**AGG**TATCTGCCGGAAGCG |
| ftsW-E289R-R | CGCTTCCGGCAGATA**CCT**CAGTTTTTGTACCGAG |
| ftsW-E289L-F | CTCGGTACAAAAACTG**CTG**TATCTGCCGGAAGCG |
| ftsW-E289L-R | CGCTTCCGGCAGATA**CAG**CAGTTTTTGTACCGAG |
| ftsW-E289Q-F | CTCGGTACAAAAACTG**CAG**TATCTGCCGGAAGCG |
| ftsW-E289Q-R | CGCTTCCGGCAGATA**CTG**CAGTTTTTGTACCGAG |
| pLY91-I-F | GG***GAATTC***GGATAAACGCGACGCATGAA |
| pLY91-I-R | CC***AAGCTT***TTACGATCTGCCACCTGTCC |
| pLY107-I-F | CGGTACCCGGGGATCC***TCTAGA***GGATAAACGCGACGCA |
| pLY107-I-R | ATCCGCCAAAACAGCC***AAGCTT***TTACGATCTGCCACCT |
| pLY123-BamHI-F | CG***GGATCC***CATCAGCAGAGTGACAG |
| pLY123-EcoRI-R | CG***GAATTC***TTATTTTTGCACTACG |
| pLY124-BamHI-F | CG***GGATCC***GGGTAAACTAACGCTGC |
| pLY124-EcoRI-R | CG***GAATTC***TTATCGATTGTTTTGCC |
| 5-EcoRI-FtsW | GACT***GAATTC***CTGGCGAAGGAGTTAGGTTG |
| 3-ftsW-XbaI | GACT***TCTAGA***TCGTGAACCTCGTACAAACG |
| 5-XbaI-ftsQ | *GAC****TCTAGA****CCAGGTTCAGCAGCCAGTGATGGATCGCTACTCGCA*  *GGCTGCTCTGAAC* |
| 3-ftsQ-EcoRI | GACT***GAATTC***TTATCATTGTTGTTCTGCC |
| 5-BamHI-ftsI | GACT***GGATCC***CAAAGCAGCGGCGAAAACGC |
| 3-ftsI-EcoRI | GACT***GAATTC***TTATTACGATCTGCCACCTG |
