# Supplementary figures and images for: Genetic analysis of the septal peptidoglycan synthase FtsWI complex supports a conserved activation mechanism for SEDS-bPBP complexes
Source: PLoS Genet. 2021 Apr 15;17(4):e1009366. doi: 10.1371/journal.pgen.1009366 (PMC8078798; doi:10.1371/journal.pgen.1009366)

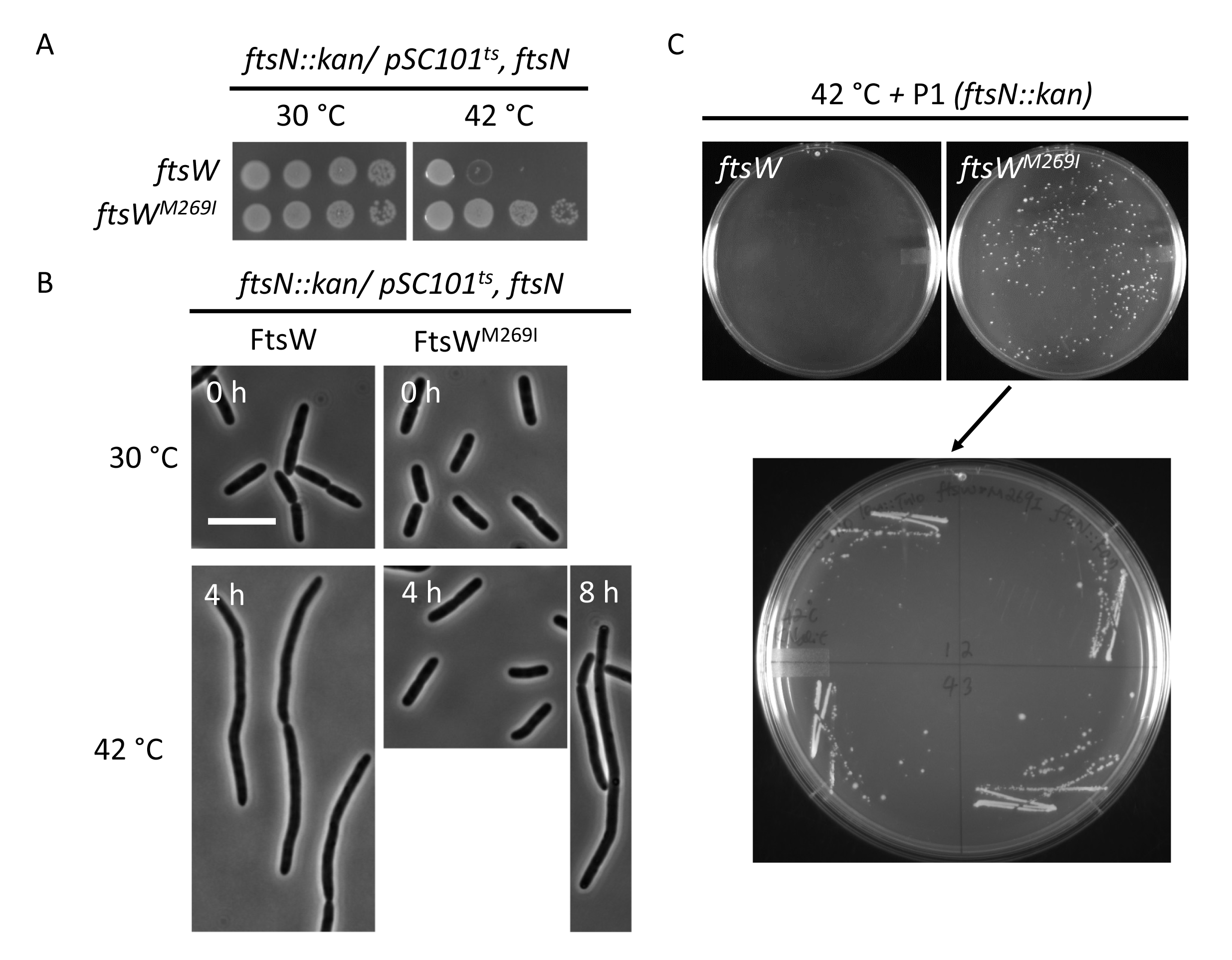

Supplement: S1 Fig — (A) Spot test of the ability of FtsWM269I to tolerate the depletion of FtsN. Colonies of strain SD264 [W3110, ftsN::kan/pBL154 (pSC101ts, Psyn::ftsN, spc)] and SD265 [W3110, ftsWM269I ftsN::kan/pBL154 (pSC101ts, Psyn::ftsN, spc)] was resuspended in 1 ml of LB medium, serially diluted. 3μl of each dilution was spot on LB plates with antibiotics. Plates were incubated at 30°C for 24 hours or at 42°C overnight and photographed. (B) Morphology of strain SD264 and SD265 at 30 and 42°C. Overnight culture of SD264 and SD265 were diluted 1:100 in fresh LB medium with antibiotics and grown at 30°C for 2 hours. Samples were taken for photograph or further diluted in 1:10 in LB medium and shifted 42°C. The cultures were kept in exponential phase and samples were taken for photograph at indicated time points after the temperature shift. (C) P1 transduction to test the ability of FtsWM269I to bypass FtsN. P1 transduction of ftsN::kan from strain CH34/pMG20 (ftsN::kan) was attempted into S3 (W3110 leu::Tn10) and SD247 (W3110 leu::Tn10, ftsWM269I) following a standard procedure. Transductants were selected on LB plates with kanamycin and 1 mM sodium citrate. Plates were incubated at 30, 37, and 42°C overnight or up to 24 hours. Obtained transdutants were restreaked on LB medium with kanamycin and 1 mM sodium citrate for further growth. Only the results from 42°C was shown. Scale bar is 5 μm. (TIF) [file pgen.1009366.s005.tif]

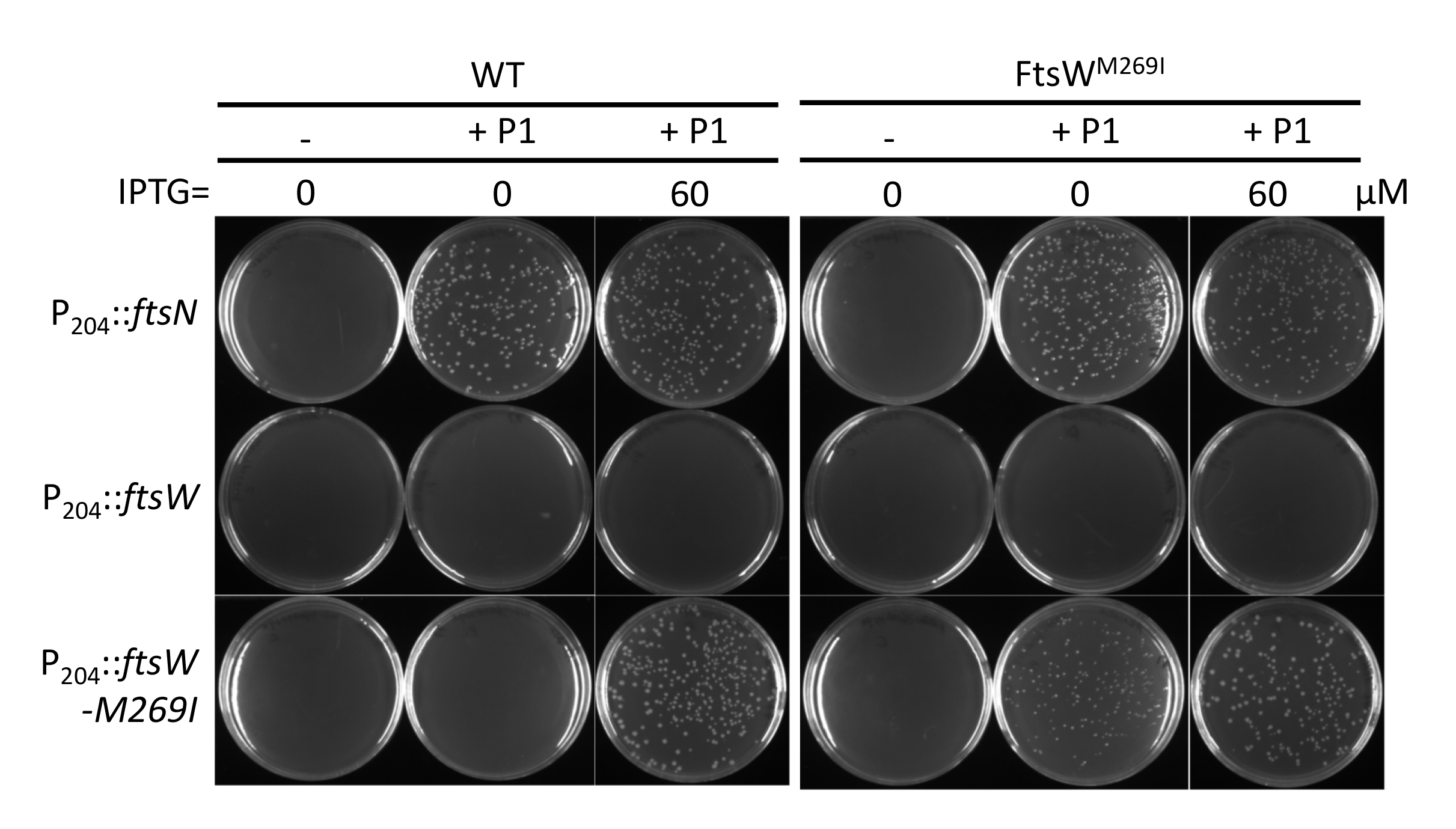

Supplement: S2 Fig — P1 transduction of ftsN::kan from strain CH34/pMG20 (ftsN::kan) was attempted into W3110 and SD247 (W3110 leu::Tn10, ftsWM269I) harboring plasmid pSEB417 (P204::ftsN), pSEB429 (P204::ftsW) or pSEB429-M269I (P204::ftsWM269I) following a standard procedure. Transductants were selected on LB plates with ampicillin, kanamycin, 1 mM sodium citrate and with or without 60 μM IPTG. Plates were incubated at 30°C up to 24 hours or 37 or 42°C overnight. Similar number of transductants were obtained for a control expressing FtsN and when FtsWM269I was induced with 60μM IPTG. Only the results from 37°C was shown. (TIF) [file pgen.1009366.s006.tif]

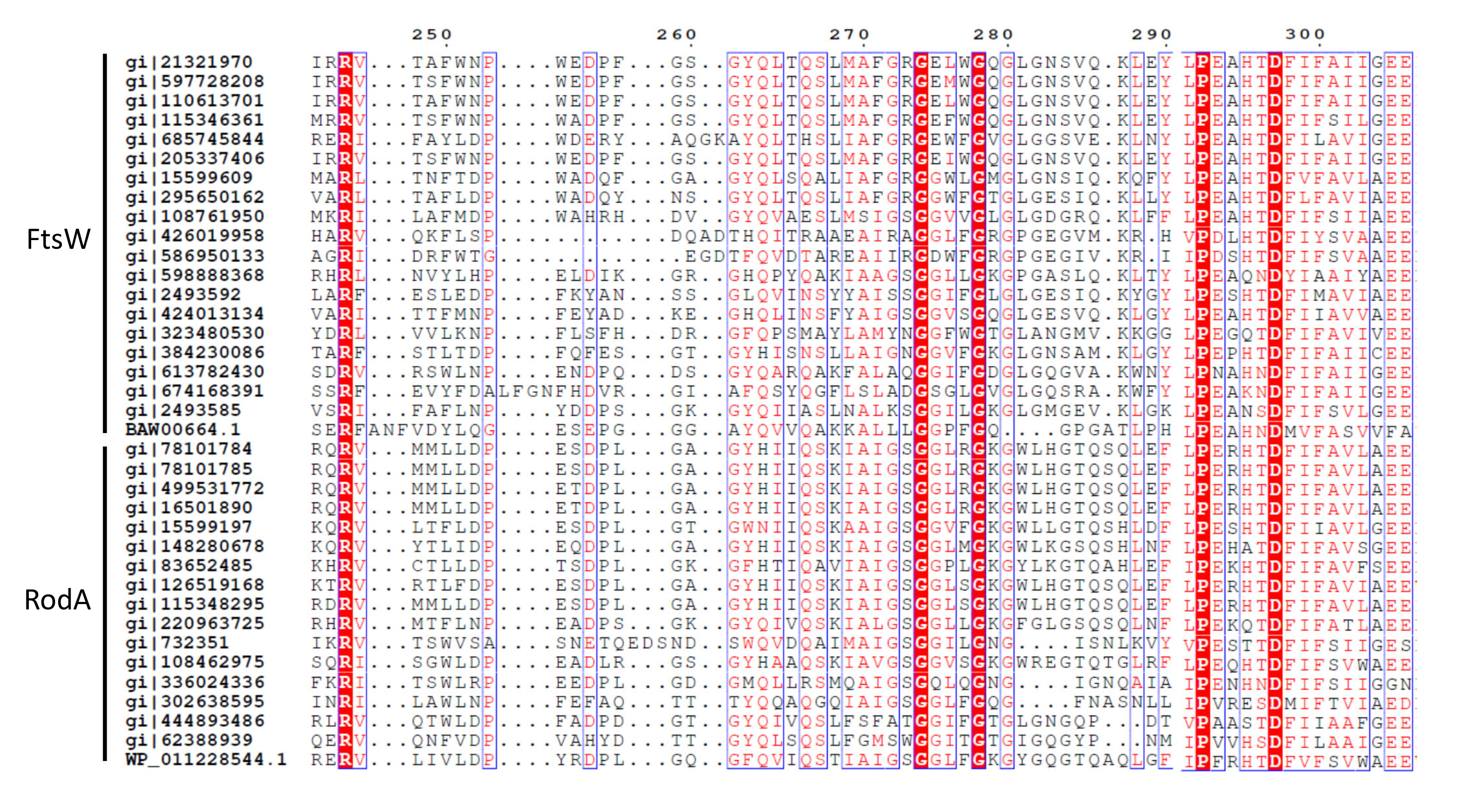

Supplement: S3 Fig — Amino acid sequences were obtained from NCBI, aligned with Clustal Omega and then created using ESPRIPT: http://espript.ibcp.fr/. FtsW: E. coli (gi|2132970), K. pneumoniae (gi|597728208), S. flexneri (gi|110613701), Y. pestis (gi|115346361), B. thailandensis (gi|685745844), S. enterica (gi|205337406), P. aeruginosa (gi|15599609), L. pneumophila (gi|295650162), M. xanthus (gi|108761950), C. crescentus (gi|426019958), A. tumefaciens (gi|586950133), B. fragilis (gi|598888368), B. subtilis (gi|2493592), L. monocytogenes (gi|424013134), E. faecalis (gi|323480530), S. aureus (gi|384230086), M. tuberculosis (gi|613782430), C. glutamicum (gi|674168391), B. burgdoferi (gi|2493585), T. thermophiles (BAW00664.1). RodA: E. coli (gi|78101784), S. flexneri (gi|78101785), K. pneumonia (gi|499531772), S. enterica (gi|16501890), P. aeruginosa (gi|15599197), L. pneumophila (gi|148280678), B. thailandensis (gi|83652485), V. cholera (gi|126519168), Y. pestis (gi|115348295), C. crescentus (gi|220963725), B. subtilis (gi|732351), M. xanthus (gi|108462975), L. monocytogenes (gi|336024336), S. pneumoniae (gi|302638595), M. tuberculosis (gi|444893486), C. glutamicum (gi|62388939), T. thermophilus (WP_011228544.1). (TIF) [file pgen.1009366.s007.tif]

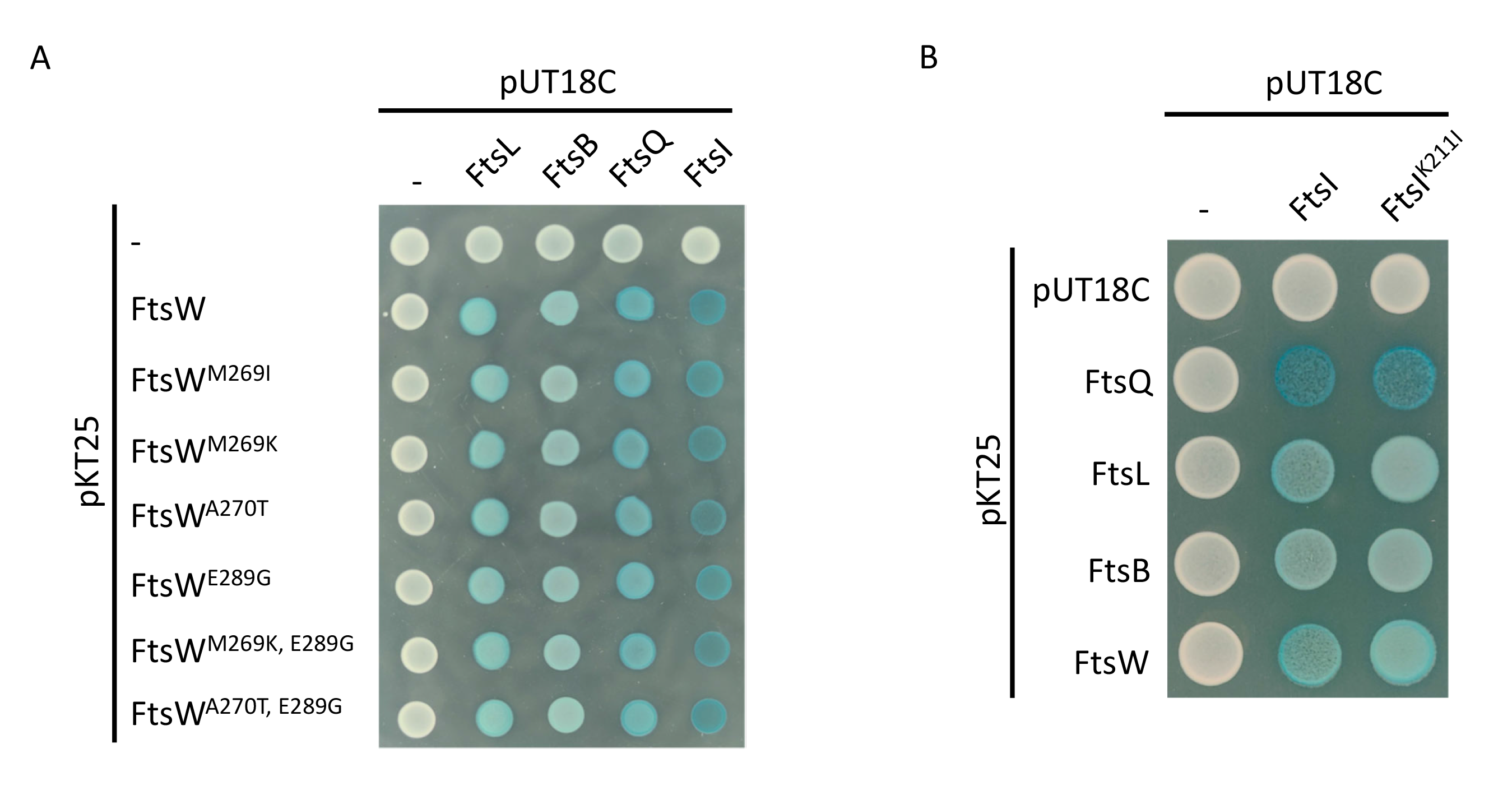

Supplement: S4 Fig — Bacterial two hybrid test of the interaction between FtsW (A) or FtsI (B) and other divisome proteins. Plasmids pairs were transformed into strain BTH101, the next day a single transformant of each resulting strain was resupended in 1 ml LB medium, 3μl of each culture was spot on LB plates containing antibiotics, 40 μg/ml X-gal and IPTG. Plates were incubated at 30°C for about 24 hours before photographing. (TIF) [file pgen.1009366.s008.tif]

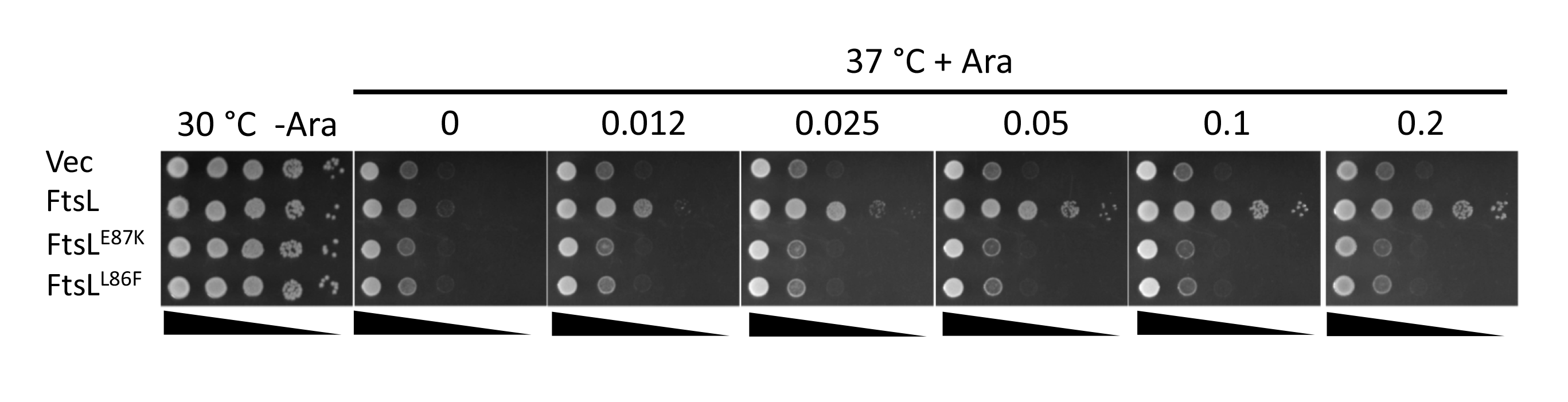

Supplement: S5 Fig — Plasmid pBAD33, or pSD296 (PBAD::ftsL) or its derivatives with different ftsL allele were transformed into strain SD399 (W3110, ftsL::kan /pSD256) harboring plasmid and transformants selected on LB plates with antibiotics and glucose. The next day, a single colony of each resulting strain was resuspended in 1 ml LB and serially diluted by 10. 3 μl of each dilution was spot on LB plates with appropriate antibiotics, with or without arabinose. Plates were incubated at 30°C for 24 hours or at 37°C overnight and photographed. (TIF) [file pgen.1009366.s009.tif]

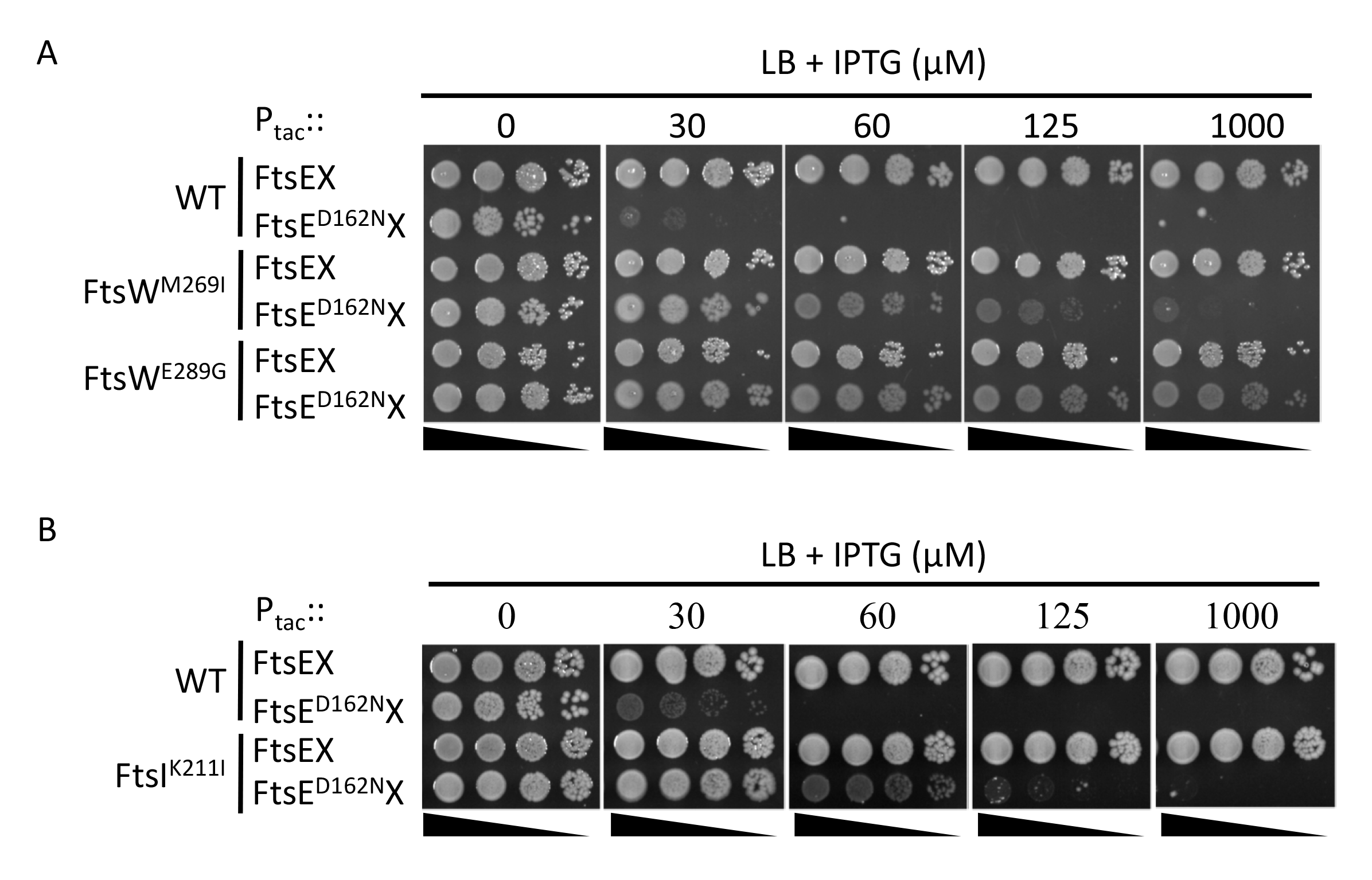

Supplement: S6 Fig — FtsWE289G (A) and FtsIK211I (B) provide resistance to the division inhibitory activity of FtsED162NX. plasmid pSD221 (pEXT22, Ptac::ftsEX) or pSD221-D162N (pEXT22, Ptac::ftsED162NX) were transformed into strain W3110, SD247 (W3110, leu::Tn10, ftsWM269I), SD488 (W3110, leu::Tn10, ftsWE289G) or LYA8 (W3110, leu::Tn10, ftsIK211I). The next day, a single transformant of the resulting strains was resuspended in 1 ml BL medium and serially diluted in 10. 3 μl of each dilution was spotted on LB plates with antibiotics and with or with IPTG. Plates were incubated at 37°C overnight and photographed. Note that the resistance of SD488 to FtsED162NX was stronger than that of strain SD247. (TIF) [file pgen.1009366.s010.tif]

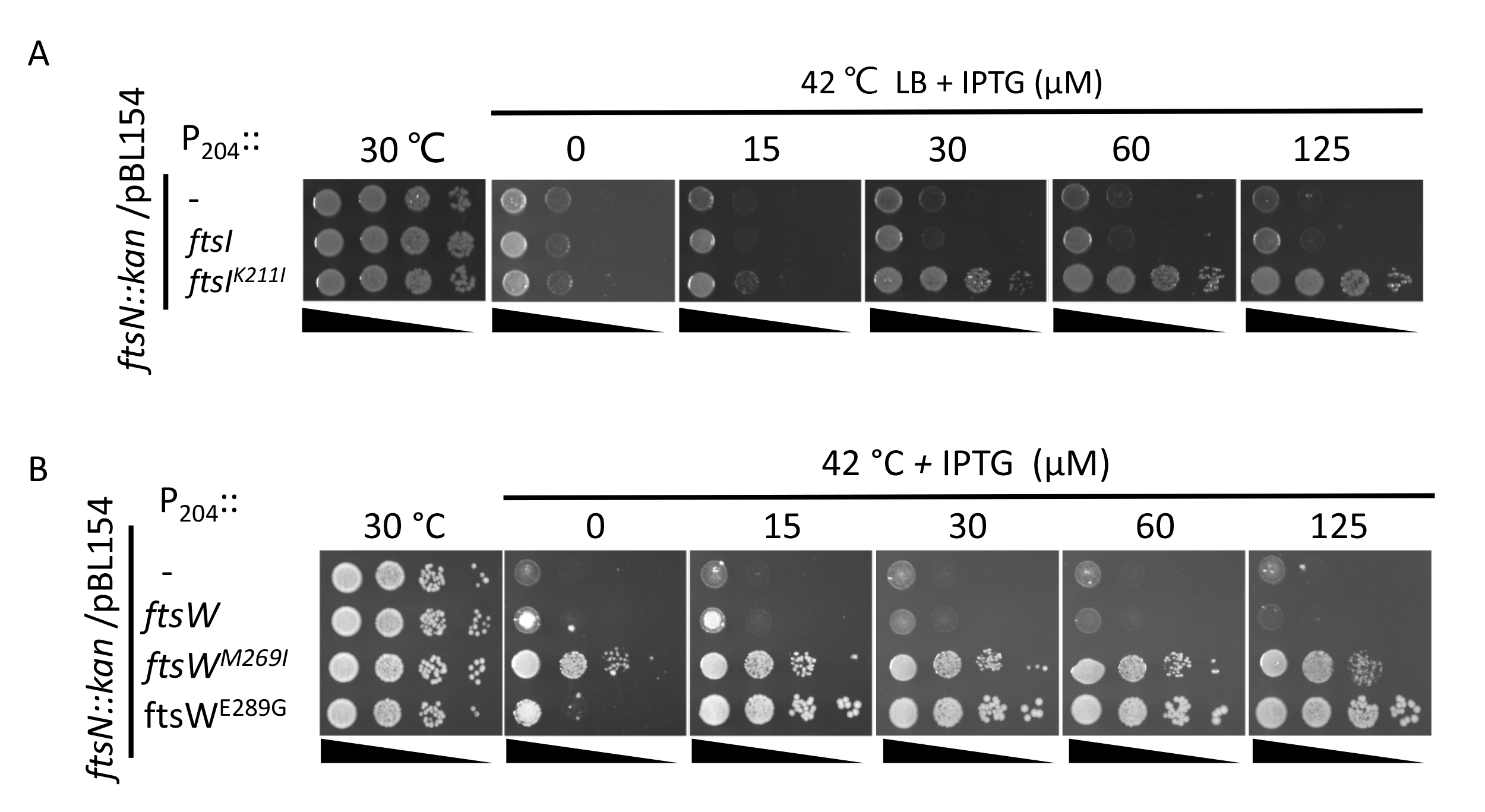

Supplement: S7 Fig — Overexpression of FtsIK211I (A) or FtsWE289G (B) tolerates the depletion of FtsN. Plasmid pDSW208, pLY91 (pDSW208, P204::ftsI), pSEB429 (pDSW208, P204::ftsW), or their derivatives carrying different ftsI or ftsW allele were transformed into strain SD264 [W3110, ftsN::kan /pBL154 (pSC101ts, ftsN)] and transformants selected on LB plates with antibiotics and glucose at 30°C. The test was performed as Fig 3B. (TIF) [file pgen.1009366.s011.tif]

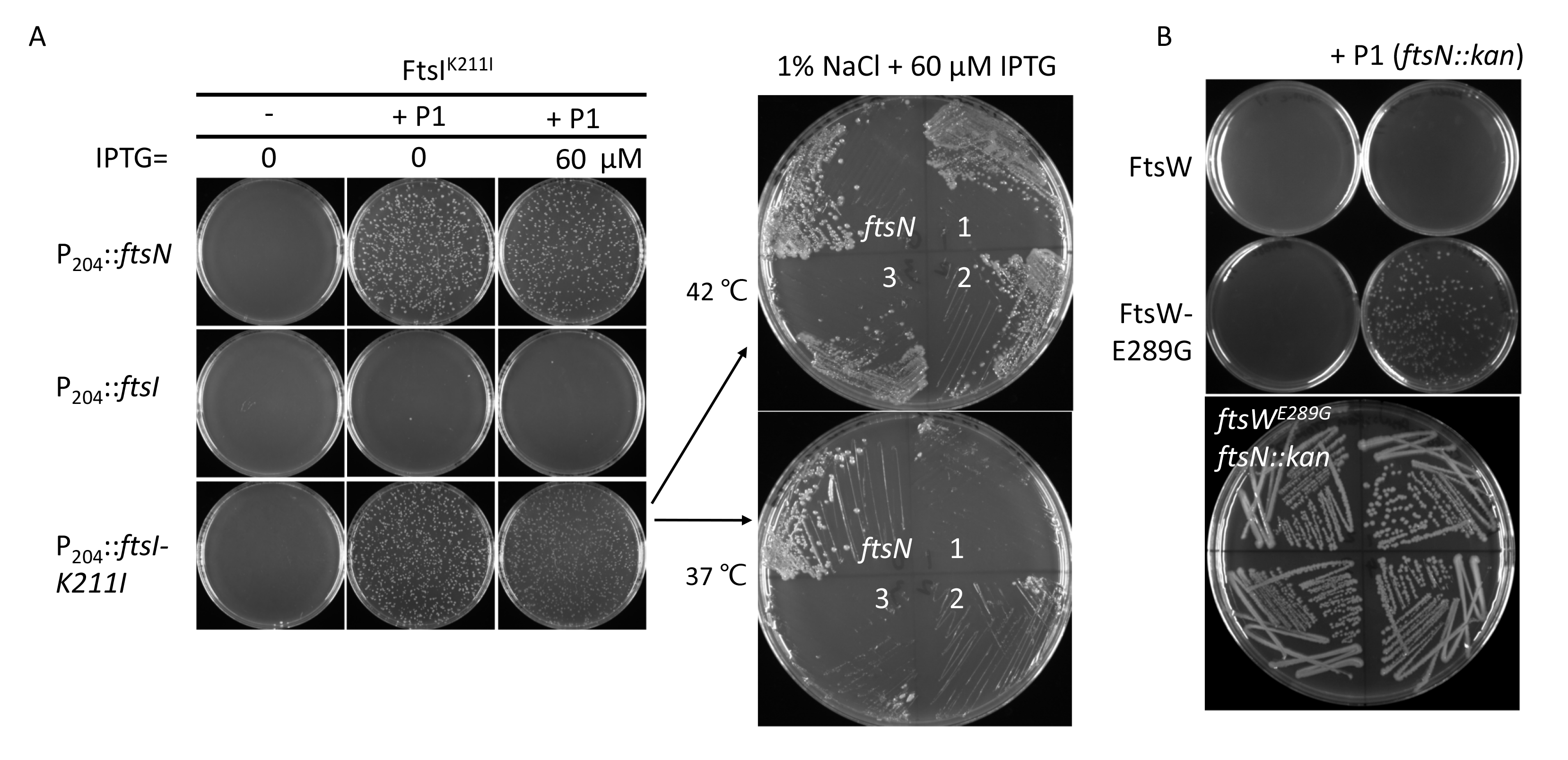

Supplement: S8 Fig — (A) P1 transduction to test the ability of FtsIK211I (A) and FtsWE289G (B) to bypass FtsN. P1 transduction of ftsN::kan from strain CH34/pMG20 (ftsN::kan) was attempted into W3110, LYA8/pLY105 [W3110, leu::Tn10, ftsIK211I / P204::ftsIK211I] and SD488 (W3110, leu::Tn10, ftsWE289G) following a standard procedure. In the case of LYA8/pLY105, transductants were selected on LB plates with 1% NaCl, kanamycin, 1 mM sodium citrate and different concentration of IPTG at 30, 37, and 42°C. Transductants were only obtained at 42°C and they can only grow at 42°C when restreaked. For SD488, transductants were selected on LB plates with kanamycin and 1 mM sodium citrate at 30, 37, and 42°C. A similar number of transductants were obtained at all three temperatures and only the result from 37°C is shown. 4 transductants from SD488 (W3110, leu::Tn10, ftsWE289G) were restreaked on the same selection plates at 37°C and all grew well. (TIF) [file pgen.1009366.s012.tif]

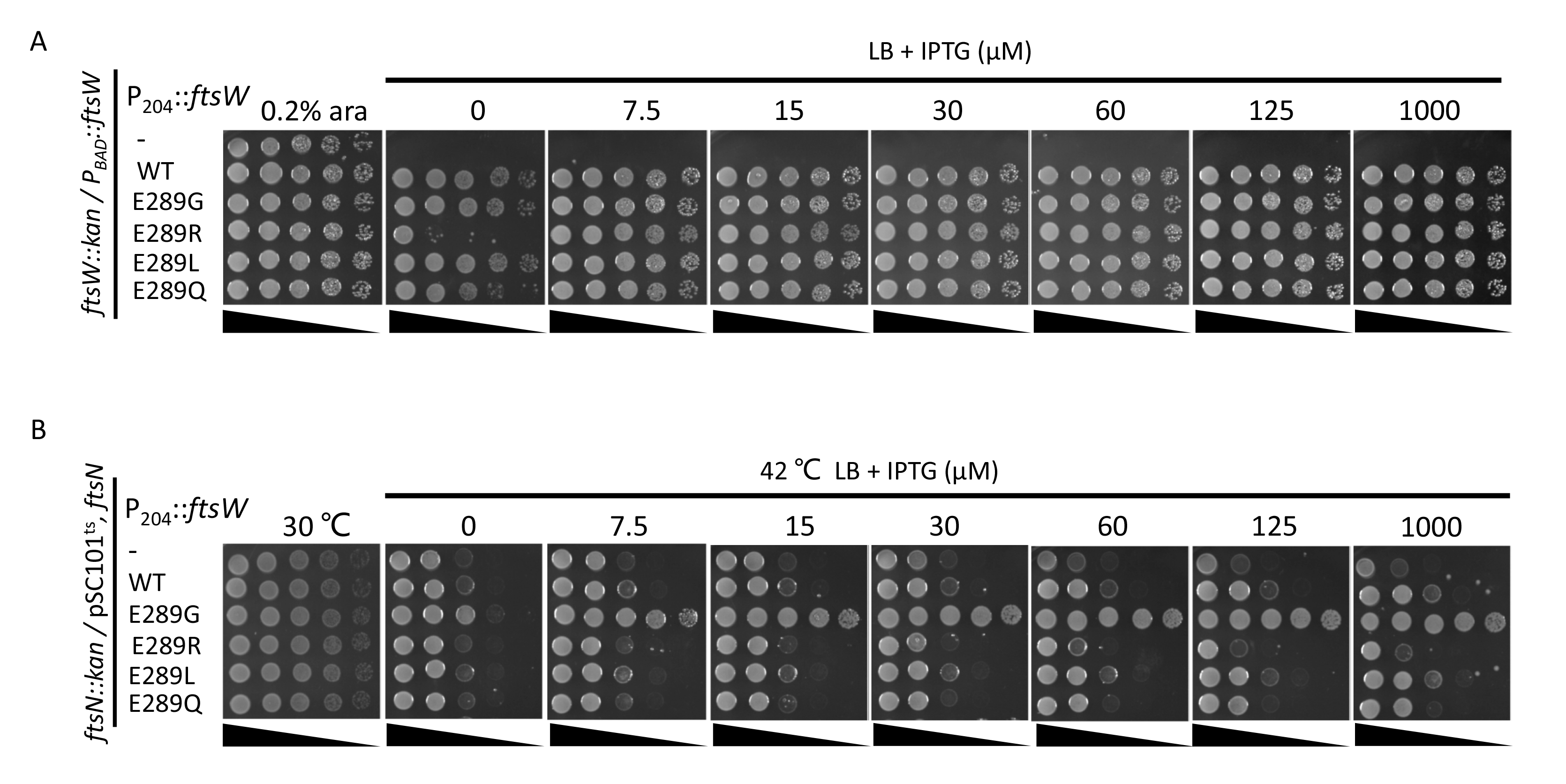

Supplement: S9 Fig — (A) Complementation test of the FtsW mutants using strain SD237. (B) Spot test of the ability of FtsW mutants to rescue the growth of FtsN depletion strain at non-permissive condition. The tests were done as in Fig 3. (TIF) [file pgen.1009366.s013.tif]

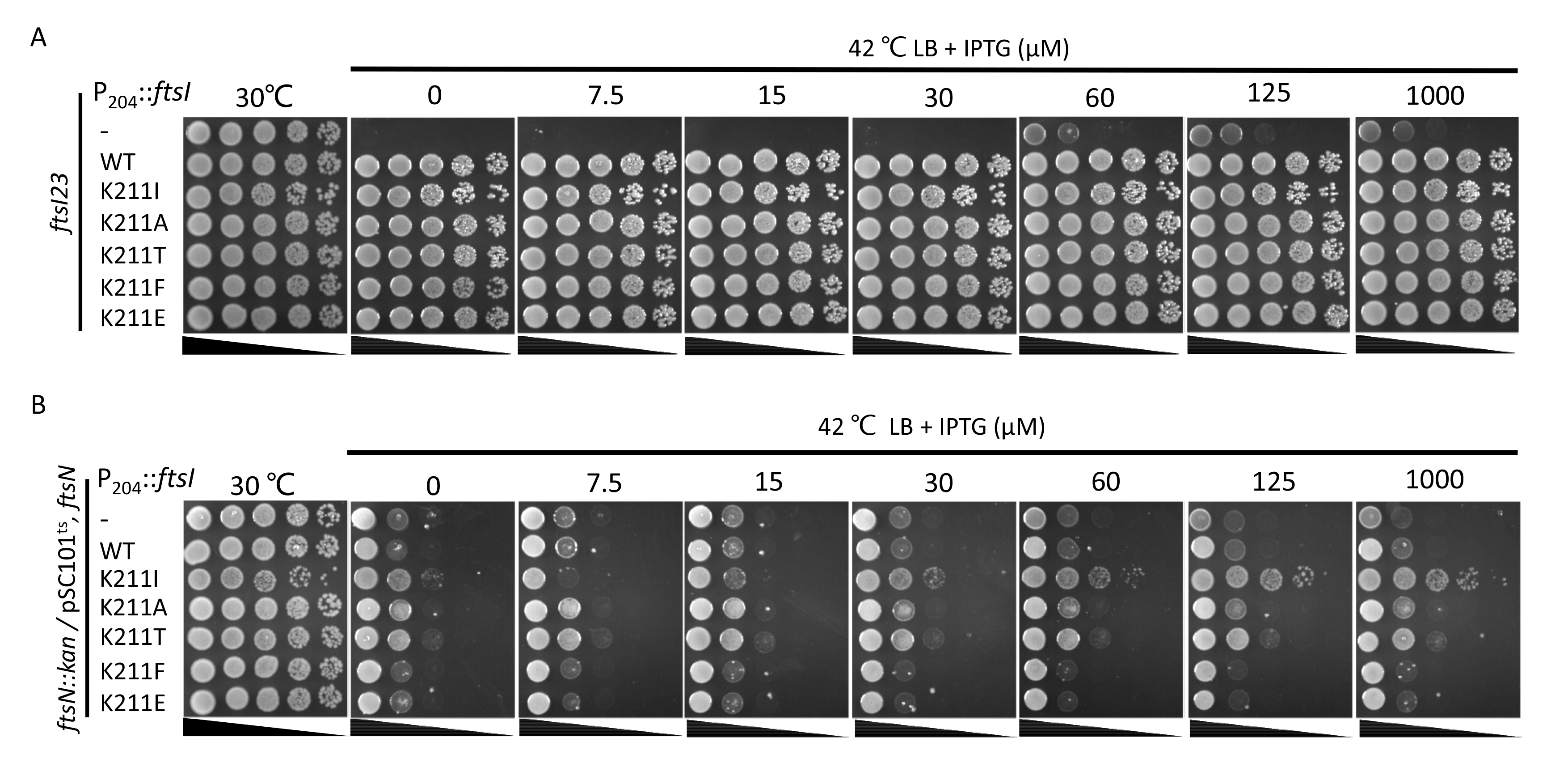

Supplement: S10 Fig — (A) Complementation test of the FtsI mutants. Plasmids pDSW208, pLY91 (P204::ftsI) and pLY105 (P204::ftsIK211I) were transformed into strain PS413 (W3110, ftsI23). A single transformant of each resultant strain was resuspended in 1 ml LB and serially diluted by 10. 3 μl of each dilution was spot on LB plates with antibiotics and with or without IPTG. Plates were incubated at 30°C for 24 hours or at 42°C overnight and photographed. (B) Spot test of the ability of FtsI mutants to rescue the growth of FtsN depletion strain at non-permissive condition. Test was done as in S7B Fig. (TIF) [file pgen.1009366.s014.tif]

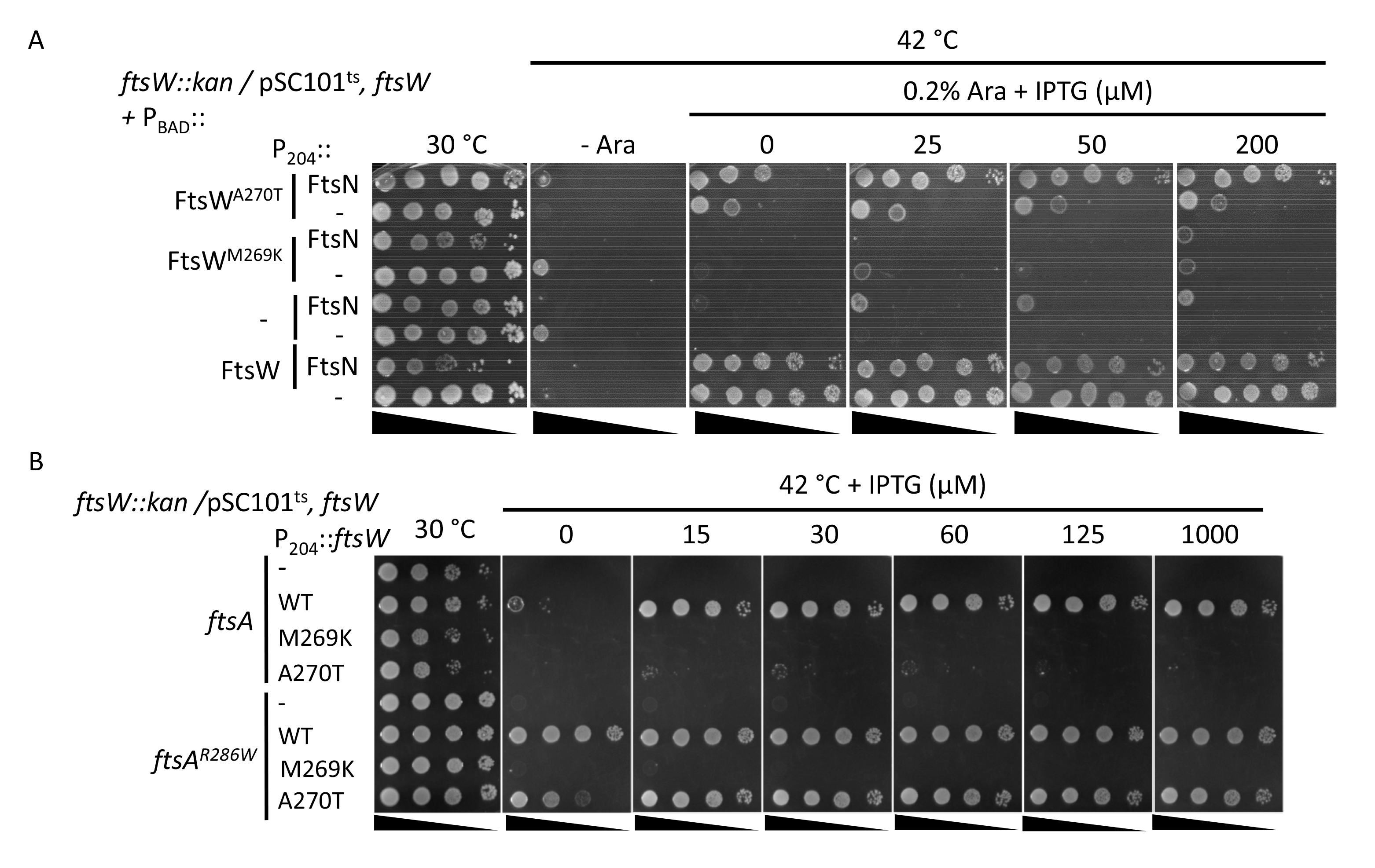

Supplement: S11 Fig — (A) spot test of the ability of FtsN overexpression to suppress FtsWA270T and FtsWM269K. (B) Spot test of the ability of FtsAR286W to suppress FtsWA270T and FtsWM269K. The test was carried out as in Fig 9 but in strain SD295 [W3110, ftsW::kan recA56 slrD::Tn10 /pSD257 (pSC101ts, ftsW)] or SD390 [W3110, ftsAR286W, ftsW::kan recA56 slrD::Tn10 /pSD257(pSC101ts, ftsW)]. Note that FtsWA270T was unable to complement the FtsW depletion strain but FtsAR286W suppressed the defect completely. In addition, in the presence of FtsAR286W, less inducer was needed to induce wild type FtsW for complementation. (TIF) [file pgen.1009366.s015.tif]
