# Supplementary material for: Genetic analysis of the septal peptidoglycan synthase FtsWI complex supports a conserved activation mechanism for SEDS-bPBP complexes
Source: PLoS Genet. 2021 Apr 15;17(4):e1009366. doi: 10.1371/journal.pgen.1009366 (PMC8078798; doi:10.1371/journal.pgen.1009366)
Supplement: Attachment — Submitted filename: Response to reviewers.docx [file pgen.1009366.s016.docx]

**Response to Reviewers’ comments**

We would like to thank the editor and the reviewers for their comments and insightful suggestions to improve the manuscript. The point-by-point response is provided below.

Shishen,

Three reviewers (and myself) were very enthusiastic about your manuscript. We found it timely, important, and well-conducted. Nonetheless, the reviewers provide good comments for you to consider as part of a revision. Nice work, and thanks for submitting to PLoS Genetics.

Dan

Response: We are glad that you liked the manuscript. We have responded to the reviews and have also edited the manuscript to make it more readable. Although not that important to this manuscript, we think the activation model (FtsN>FtsQLB-FtsL> FtsI>FtsW) nicely explains why the FtsI homolog (PBP2B) in *B. subtilis* is essential even though the transpeptidase activity is not. FtsI (PBP2B) is needed for the signal relay and another PBP (PBP3 in *B. subtilis*) can provide the transpeptidase activity needed to crosslink the glycan strands synthesized by FtsW. *E. coli* does not have that back up PBP and instead makes uncrosslinked glycan that is degraded by Slt [futile cycle]). It is also nice that the activation mechanism for FtsW-FtsI is similar to RodA-PBP2 despite the regulators being completely unrelated.

Reviewer #1: This manuscript by Li et al provides significant further genetic evidence and molecular details supporting a model of interaction/ activation of SEDS family glycosyltransferase FtsW by its class b PBP partner FtsI during E. coli cell division. They further characterize a previously isolated activation mutant of FtsW (M269I) and isolate additional mutants of FtsW (E289G) and FtsI (K211I) that further clarify a main pathway of activation of E. coli divisome peptidoglycan synthesis (FtsN --> FtsL (in association with FtsQB) --> FtsI --> FtsW), with FtsA and FtsEX also figuring into this regulated septal cell wall synthesis. The authors do an excellent job of using genetics to tease out interactions between all of these players and a hierarchy of their activities, building/confirming existing models proposed for the SEDS/bPBP partner interactions in cell length growth (RodA/PBP2). Consideration of their results when mapping mutations onto RodA/PBP2 structure interactions likely analgous to FtsW/FtsI reveal that activation of FtsW through FtsI occurs via the extracellular loop 4 (ECL4) of FtsW with the 'pedestal domain' of FtsI, separate from regions responsible for recruiting these proteins to the divisome. At the same time, their results demonstrate that the specific residues within these domains that are most integral for activation show some variation between species.

Overall I believe the manuscript is solid, with conclusions well-supported by the data. I only have some minor comments:

Response: We thank the reviewer for the comments and suggestions to improve the manuscript.

- The initial results with the A270T mutation of FtsW are interesting and also become important for later portions of the paper in that it serves as a partially 'inactive' form. This is stated within lines 172 - 173, with reference to figure SF4, but seems a foundational piece of data that bears more stressing and explanation here. I would actually recommend that SF4 be made a regular figure given the importance of A270T later and that its 'defect' is otherwise not apparent to the reader.

Response: We thank the reviewer for this suggestion. Fig. S4 is now included as a regular figure (Fig. 4) in the revised manuscript. Numbering of the regular and supplemental figures thus have been changed accordingly.

- The caveat to lines 174 and 175 would be that the gfp adds to stability compared to a non-fused version of the mutants. The 'inactivity' of these even with the gfp though would be consistent with their statements/arguments. I would just clarify.

Response: In the western blot, we were using anti-GFP antibody to access the stability of the GFP fusions of the FtsW mutants. The first lane was actually a control expressing just GFP, the level of the FtsW mutants was comparable to WT. The sentence has been changed to “Western blot of GFP fusions of FtsW^M269K^ and FtsW^A270T^ using anti-GFP antibody showed that they were expressed at the same level as wild type FtsW (Fig. 4B), indicating that it was unlikely because they were unstable.” (line 175-177)

- The cells carrying ftsN::kan in Figure 2 panel B seem to be mostly lysing (pock-marked). Does the necessity of antibiotics here make this more prevalent perhaps? Doesn't seem to fully 'suppress' PG defects then?

Response: Antibiotics didn’t make a difference, it is just a selection marker to delete *ftsN*. FtsW^M269I^ cannot fully suppress the division defect caused by deletion of *ftsN*, which was exactly what we were trying to show in Fig. 2. Overexpression of FtsW^M269I^ can rescue the growth the *ΔftsN* cells, but the cells still have a division deficiency (longer and chainy). However, in the absence of inducer to overexpress FtsW^M269I^, *ΔftsN* cells stop dividing and lysed.

- Has an M269L allele been attempted, would they predict that might act like the Ile version?

Response: Thanks for the suggestion. We went on to test an M269L mutant, it is functional in a complementation test but does not suppress FtsN depletion. So it does not act like M269I. Since our main interest here was to see if a dramatic change would produce a more active allele, we didn’t include this result in the revised manuscript.

- Line 259. All substitutions affect protein structure by definition. Just add something like 'substantially'.

Response: Thanks for the suggestion, this was added. (line 262 in the revised version)

- Lines 262 - 264, it was unclear to me initially why one would necessarily predict this exactly. This may be in part due to my still not grasping at this point what the 'defect' with A270T was.

Response: Sorry for the confusion here. We were thinking that A270T was weaker than M269K so it was more likely to be suppressed, but it is not necessary the case. We agree with the reviewer on this and have changed the wording. (line 265-267 in the revised version)

- In Figure 4 the GFP-FtsW and GFP-FtsI signal in the M269K background seems to be infrequent and particularly irregular. Is this really still able to localize to the divisome, or are we seeing residual preformed rings that never disassembled as FtsW was depleted (maybe still containing some residual W?) The GFP-FtsW label in panel A should also be put in.

Response: We have a lot of experience with this and find that this is very typical. Other labs observed this as well, for example in (Marmont LS, Bernhardt TG. 2020 PNAS) and in (Pogliano J, et al. 1997 PNAS). Although division proteins localize to potential division sites in filaments, the number of Z rings/septal rings per unit cell length is low. This is in part due to the oscillation of Min proteins that regulates the position of the division machinery.

An FtsW-GFP label was added to panel A of Figure 4 (Figure 5 in the revised version)

- The text needs editing in terms of putting in commas. Several sentences are awkward or confusing without commas properly separating clauses, etc.

Response: Thanks for the suggestion, we have edited the manuscript with those comments in mind.

Reviewer #2: This paper from Li and co-workers investigates the activation of cell wall synthesis within the divisome by the FtsWI synthase complex. The authors use an elegant series of genetic experiments to show that amino acid substitutions in the pedestal domain of FtsI and extracellular loop 4 of FtsW can either lead to hyperactivation of the synthase or its inactivation. Epistasis studies provided additional support for the pathway of activation within the divisome triggered by FtsN flowing from FtsQLB to FtsI to FtsW. Mapping of the activating and inactivating changes in FtsWI to the structure of the related SEDS-bPBP cell wall synthase RodA-PBP2 from the elongation machinery provides evidence for a conserved activation mechanism between the two synthases as changes that activate RodA-PBP2 map to the same region of the structure. Overall, this is a very nice paper that makes an important contribution to our understanding of how cell wall synthesis is activated within morphogenic machines. I have only minor points to be addressed.

Response: We thank the reviewer for the comments and suggestions to improve the manuscript.

1) Line 67: evidences are > evidence is

Response: Change made as suggested. (line 67 in the revised version)

2) Line 90: ...AWI domain of FtsL is LIKELY TO BE exposed...

Response: Thanks for the suggestion, “likely to be exposed…” is more accurate. Change made as suggested. (line 91 in the revised version)

3) Line 244-245: Thus, these two mutations do not... I think this statement is too strong since it is based solely on two-hybrid analysis. Please moderate it.

Response: We agree with the reviewer for this. We added “are unlikely to” (line 245-246 in the revised version)

4) Line 271: ...the weak mutant... change to ...the weakly defective mutant...

Response: Thanks for the suggestion, change made. (line 274-276 in the revised version)

5) Line 273: Similarly, it would be better to describe the other mutant as the "severely defective mutant".

Response: Thanks for the suggestion, change made. (line 274-276 in the revised version)

6) Line 287: Change "inactive mutations" to "inactivating mutations".

Response: Thanks for the suggestion, change made. (line 287 in the revised version)

7) Line 307: ...whether THEY are...

Response: Thanks for the suggestion, change made. (line 311 in the revised version)

8) Table 1: The text is not colored as indicated in the legend.

Response: Sorry for our carelessness. The text was now colored as indicated. (Page 18)

9) Line 336: ...resulting in... is too strong. Change to "likely causing".

Response: Thanks for the suggestion, change made. (line 343 in the revised version)

10) Line 410: I think you mean POSITIVE charge of K211

Response: Sorry for our carelessness, changed made. (line 423 in the revised version)

11) Some of the spot assay figures could be better labeled to make it easier for the reader to follow. For example, Fig. 5A, it is not clear from looking at the figure that FtsL is depleted by a temperature shift. Please review and modify figures accordingly to make them easier to follow.

Response: Thanks for the suggestion, the genotype of the strains were now indicated in the Fig. 5A as well as other others.

Reviewer #3: 1. Could the authors clarify the relationship between two possible activation pathways, one form FtsN, and one from FtsEX, on the activation of FtsW? Are they independent and redundant of each other? FtsW superfission mutants M269I and E289G can suppress FtsE(D162N)X or FtsN depletion separately. Can they suppress both at the same time (FtsE(D162N)X + FtsN depletion)?

Response: Thanks for the question. FtsEX does not initiate an activation pathway of FtsWI, it is a part of the activation pathway triggered by FtsN. In the periplasm, FtsN contacts FtsQLB, likely causing a conformational change in FtsQLB that leads to FtsL acting on FtsI to activate FtsW. In the cytoplasm, FtsN interacts with FtsA, likely causing a conformational change as well, to cause the activation of FtsWI by a yet unknown mechanism. FtsEX is needed to initiate recruitment of FtsQLBWI and must hydrolyze ATP for the activation of FtsWI to occur. If FtsEX does not hydrolyze ATP, activation is blocked. It is likely that the activation signal from FtsA is blocked in the absence of ATP hydrolysis by FtsEX, but the details still need to be worked out.

We have not tested if FtsW^M269I^ and FtsW^E289G^ can suppress both FtsE^D162N^X and FtsN depletion simultaneously, but I guess the stronger FtsW^E289G^ mutant may be able to do it because it suppresses FtsN deletion so well and provides good resistance to FtsE^D162N^X. We are actually trying to use these activating mutations to bypass multiple regulatory proteins in this activation pathway, stay tuned.

2. I am not sure about the statement that the transpeptidase activity of FtsI is not essential, Antibiotics such as Aztreonam specifically inhibits FtsI’s transpeptidase activity leads to the cease of cell division, long filamentation, and eventually death. I think I understand the authors’ point of view that another essential function of FtsI is to activate FtsW, for which FtsI’s transpeptidase activity is not required. This point should be clarified.

Response: We apologize that we didn’t explain clearly enough in the manuscript. The transpeptidase activity of FtsI is essential in E. coli and many other bacteria. However, in *Bacillus. subtilis* the transpeptidase activity of the FtsI homolog (PBP2B) can be clearly bypassed even though the protein has to be present. In this case another PBP (PBP3) crosslinks the PG glycan strands synthesized by FtsW, which requires PBP2B for recruitment to the division site and becomes essential when the transpeptidase activity of PBP2B is blocked (Ref 40, Sassine, et al). This is also the case in *S. aureus* (Reference 41, Reichmann et al.). Apparently, in Gram positive bacteria there is a backup PBP that can do the job, but this PBP requires the presence of FtsI, likely because FtsI is needed to activate FtsW.

The paragraph discussing this point was re-written. (line 379-394 in the revised version)

3. The authors’ observations that the negative charges of E289 and K211 are not essential are interesting. They imply that the interactions between FtsW and FtsI may not be typical electrostatic protein-protein interactions. Could the authors offer more specific insight regarding how this could be possible based on the structural model, other than “subtle local re-arrangements of the FtsWI complex”? for example, some type of structural model?

Response: The residue corresponding to E289 is in a region of RodA that is unstructured in the RodA-PBP2 structure so it is a little premature to say. What it suggests is that the side chain is important and removing it locks FtsW in the active conformation. Perhaps the interaction with FtsI causes a change in conformation in the side chain activating FtsW.

4. I am not sure about the epistatic analysis of FtsW-E289G on M269K and A270T. To me it is an effect of E289G overwriting M269K and A270T, but this overwriting function is not dependent on the presence of M269K or A270T. In other words, the super activation phenotype of W289G is not dependent on M269K or A270T, as it can compensate FtsW depletion. M269K reduced the activity of E289G, certainly indicating some type of interactions between the two sites but does not mean that one mutant is dependent on the other to function. Or am I understand the concept of epistasis in the wrong way?

Response: We agree with your interpretation. E289G results in a super active allele that bypasses the other mutations. In other words any input from what is happening at M269 is no longer required.

Minor comments:

1. Lines 110-124: Including clarification in the text to Figs. S1A and S1B will allow readers to better match the text and figure for a better understanding.

Response: Thanks for the suggestion. This was added. (line 117 and line 122)

2. Lines 276-296: Including clarification in the text to Figs. 9A and 9B will allow readers to better match the text and figure for a better understanding.

Response: The text was clarified by adding 10A and 10B (originally Fig 9A and 9B) to the text along with a few changes to the text and figure legend.

3. Page 17: In the Table 1 description it lists “Active (green)” however there is no green text in the table or text.

Response: We apologize for our carelessness. This was corrected to be compatible with Fig. 11 in the revised manuscript (originally Fig. 10)

4. Figure 10 (structure with mutations) and Table 1 (mutations) are related, but minor changes will make the connection stronger. Figure 10 has increased activity mutations in orange, inhibiting mutations in magenta, and the catalytic residue in red. Table 1 has active mutations in green. Keeping a consistent color scheme between the structure figures and the table would be helpful.

Response: Thanks for the suggestion. This was corrected so that the Table 1 and Fig. 11 (originally Fig. 10) are compatible.
